# Relay-MSRE-qPCR: A sequential multi-buffer staged-dilution workflow for methylation-sensitive restriction enzyme qPCR

**DOI:** 10.1016/j.mex.2026.104205

**Published:** 2026-10-12

**Authors:** Ippei Uemura, Takashi Satoh, Natsuko Takahashi-Suzuki

**Affiliations:** Department of Pharmaceutics, Faculty of Pharmaceutical Sciences, Hokkaido University of Science, 7-Jo 15-4-1 Maeda, Teine-ku, Sapporo, Hokkaido, 006-8585, Japan

**Keywords:** MSRE-qPCR, DNA methylation, CpG islands, Epigenetic analysis

## Abstract

Methylation-sensitive restriction enzyme quantitative PCR (MSRE-qPCR) quantifies locus-specific DNA methylation in native DNA without bisulfite conversion. However, such multi-enzyme workflows are limited by buffer incompatibility, cumulative inhibitor carry-over, and sample loss during purification. We developed Relay-MSRE-qPCR, a sequential staged-dilution workflow in which each heat-inactivated digest is transferred to a fresh tube, diluted ≥5-fold to attenuate inhibitors, and then the buffer for the next enzyme is reconstituted to 1 × concentration. Buffer budgeting kept carry-over compatible with qPCR, with ≤5% (v/v) glycerol included per step. With Eco52I (XmaIII), HhaI, EcoRII, VpaK11BI (AvaII), and HpaII, the relay maintained restriction-site-responsive digestion and the cumulative amount of salts, Mg²⁺, and stabilizers remained within polymerase-tolerable limits; defined unmethylated PCR-product controls showed marked loss of amplifiable template for all five enzymes. Relay and single-enzyme comparator conditions showed consistent delta cycle threshold (ΔCt) behavior, and qPCR products exhibited single-peak melt curves under the tested conditions. Only enzymes recognizing a site within the PCR amplicon produced ΔCt shifts, confirming site-specific responsiveness. A practical protocol is provided with guidance for amplicon placement, enzyme selection, and buffer accounting. Relay-MSRE-qPCR enables reproducible, low-cost targeted CpG methylation analysis when base-resolution sequencing is unnecessary or impractical.

Sequential staged dilution preserves enzyme-specific digestion conditions without inter-step purification.

Reduces inhibition and enhances reproducibility by budgeting carry-over components within accepted qPCR tolerance ranges.

Site-specific ΔCt shifts support reliable targeted CpG methylation analysis.

## Graphical abstract

**Figure:**
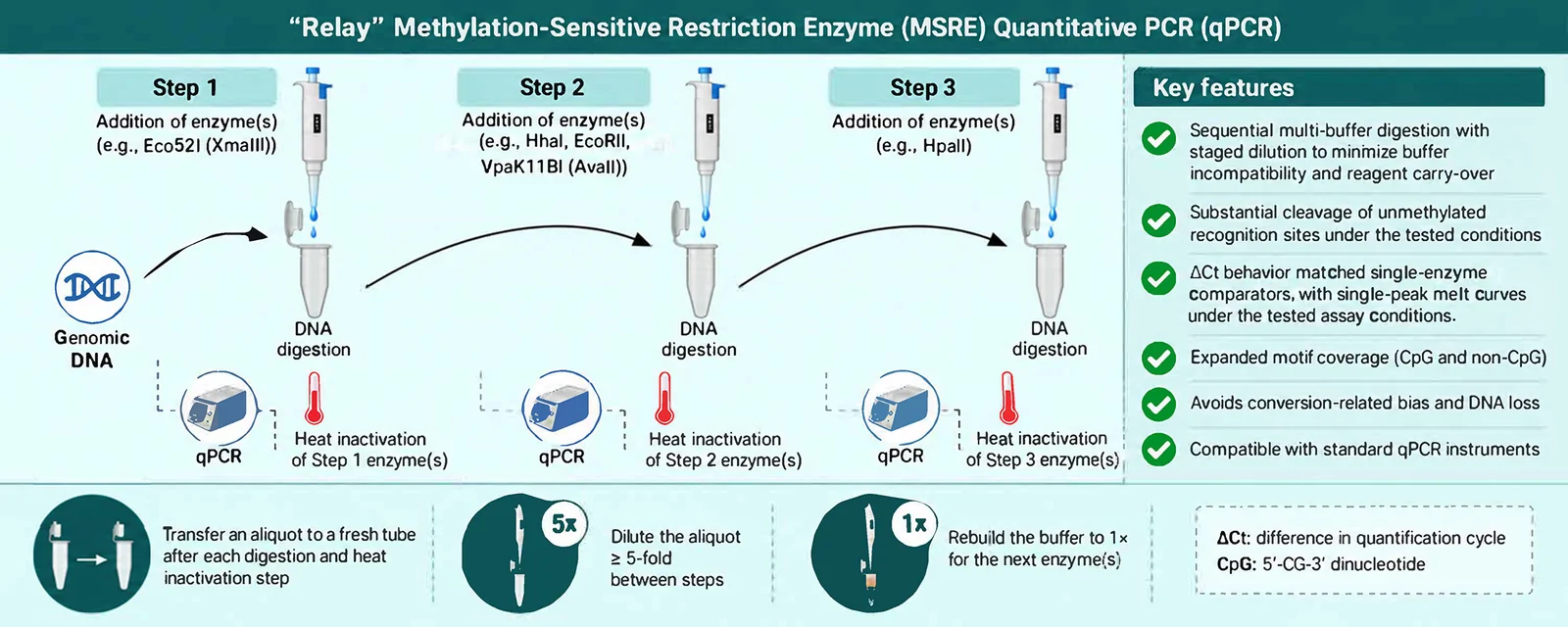

Specifications table

**Table:** 

**Subject area**	Biochemistry, Genetics and Molecular Biology
**More specific subject area**	DNA methylation analysis; qPCR assay development
**Name of your method**	Relay-MSRE-qPCR (sequential multi-buffer digestion with direct qPCR)
**Name and reference of original method**	Builds on MSRE-qPCR and sequential restriction digestion; staged buffer management without purification
**Resource availability**	Standard restriction enzymes and 10 × buffers; thermocycler/qPCR

## Background

Methylation-sensitive restriction enzyme quantitative PCR (MSRE-qPCR) quantifies locus-specific DNA methylation in native DNA by comparing delta cycle threshold (ΔCt) values between digested and undigested aliquots at methylation-sensitive sites. For example, at the CCGG motif, HpaII is blocked by methylation of the inner cytosine (CmCGG), whereas its isoschizomer MspI is insensitive to inner-CpG methylation but is blocked by methylation of the outer cytosine (mCCGG) [1,2]. Results may be reported as ΔCt or converted to percent methylation using fully methylated and unmethylated controls in accordance with the Minimum Information for Publication of Quantitative Real-Time PCR Experiments (MIQE) guidelines [3]. This targeted approach is well suited for predefined CpG islands or when DNA input is limited.

Bisulfite-based sequencing enables base-resolution analysis but can damage DNA and reduce library complexity, and requires upstream conversion and downstream bioinformatics [4,5]. Although enzymatic or chemical conversion approaches reduce some of these liabilities, they still require multi-step processing and specialized reagents that may not be optimal for small or iterative locus panels [6]. Single-enzyme MSRE assays avoid chemical conversion but often require splitting samples across motifs or performing simultaneous co-digestion in suboptimal compromise buffers, risking reduced enzyme activity and increased star activity when ionic strength, Mg²⁺ concentration, pH, or stabilizers are mismatched [2,7]. Thus, a practical gap remains for workflows that (i) retain closed-tube native-DNA qPCR, (ii) expand motif coverage without inflating input or run count, and (iii) maintain each enzyme near optimal buffer conditions. While conversion-based sequencing interrogates cytosines genome-wide, Relay-MSRE-qPCR focuses on predefined GC-rich regions and CpG islands where restriction motifs are present. Motif-free loci and true base-resolved mapping remain domains for bisulfite or enzymatic-conversion sequencing, whereas relay MSRE assays can evaluate non-island CpGs when a suitable motif lies within the amplicon. Overall, the relay complements rather than replaces conversion approaches [2,[4], [5], [6]].

We selected a five-enzyme panel to span CpG-dependent and auxiliary non-CpG contexts while stress-testing buffer transitions: HpaII (CCGG) and HhaI (GCGC) interrogate CpG-dependent 5-mC; Eco52I (XmaIII; CGGCCG) targets CpG-dense hexamers; and EcoRII (CCWGG) and VpaK11BI (AvaII; GGWCC) report methylation in non-CpG contexts [2,7]. Although non-CpG methylation is lower in most somatic epithelia, these motifs remain common in GC-rich regions, including CpG islands and shores, providing complementary sentinels [8]. Importantly, these enzymes have distinct optimal buffer requirements; neither splitting input across tubes nor co-digestion in one compromise buffer adequately preserves both efficiency and specificity [2,7,9]. The relay architecture—digestion → heat inactivation → ≥5-fold staged-dilution → rebuild the next buffer to 1 × —allows each enzyme to operate near its intended ionic and cofactor conditions while preventing cumulative inhibitor carry-over (salts, Mg²⁺, glycerol, and stabilizers). This is particularly important when combining CpG-dependent enzymes (HpaII, HhaI, or Eco52I) with enzymes that have distinct buffer requirements, including EcoRII and VpaK11BI [2,7]. The staged-dilution design preserves per-enzyme fidelity and quantitative behavior without increasing input needs or workflow complexity, aligning with MIQE-style acceptance criteria and inhibitor-avoidance thresholds [3,[10], [11], [12], [13]].

To address these constraints, we developed Relay-MSRE-qPCR, a sequential staged-dilution workflow. After each digestion and heat inactivation, an aliquot is transferred to a fresh tube, diluted ≥5-fold to attenuate carry-over (salts, Mg²⁺, and glycerol), and then the buffer of the next enzyme is restored to 1 × . This stepwise rebuild design maintains per-enzyme fidelity while keeping cumulative carry-over within qPCR-tolerable ranges, as supported by ΔCt controls and single-peak melt-curve behavior [3,7,10]. Although readouts remain motif-dependent and influenced by sequence context, the approach provides a practical balance between discovery-grade sequencing and routine closed-tube quantification—especially for hypothesis-driven panels and limited-input samples [[2], [3], [4], [5], [6]].

## Method details

### Chemicals

HpaII, HhaI, VpaK11BI (AvaII), and Eco52I (XmaIII), together with 10 × Universal Buffers L, M, and H and the Eco52I Basal buffer, were purchased from Takara Bio Inc. (Shiga, Japan). EcoRII (Code No. 315-00882) and the M buffer supplied with it were purchased from NIPPON GENE Co., Ltd. (Toyama, Japan). KAPA SYBR® FAST qPCR Master Mix was obtained from NIPPON Genetics Co., Ltd. (Tokyo, Japan). SAFELOOK™ Pre-Red pre-loading gel stain (1 × final concentration in samples) and molecular-biology-grade agarose were obtained from FUJIFILM Wako Pure Chemical Corporation (Osaka, Japan). ISOGENOME reagent was obtained from NIPPON GENE Co., Ltd. (Toyama, Japan). Ultrapure, nuclease-free water was used throughout. Reagents were stored and handled according to manufacturers’ instructions. The glycerol concentration was maintained at ≤5% (v/v) per digestion step.

### Cell culture and genomic DNA isolation

Caco-2 human colorectal epithelial adenocarcinoma cells were obtained from the European Collection of Authenticated Cell Cultures (Salisbury, UK). Cells used for the present validation were at passages 20-31 and were maintained in D-MEM (High Glucose) with L-glutamine, phenol red, and sodium pyruvate (Cat. No. 043-30085; FUJIFILM Wako Pure Chemical Corporation, Osaka, Japan), supplemented with 10% fetal bovine serum (Serana Europe GmbH, Pessin, Germany), 1% Penicillin-Streptomycin Solution (× 100; Cat. No. 168-23191; FUJIFILM Wako Pure Chemical Corporation; final concentrations, 100 U/mL penicillin and 100 μg/mL streptomycin), 1% MEM Non-essential Amino Acids Solution (× 100; Cat. No. 139-15651; FUJIFILM Wako Pure Chemical Corporation), and an additional 2 mM L-glutamine supplied from 200 mM L-Glutamine Solution (× 100; Cat. No. 073-05391; FUJIFILM Wako Pure Chemical Corporation). Cells were maintained at 37°C in a humidified atmosphere containing 5% CO₂, and the medium was replaced every 1-2 days. At approximately 100% confluence, cells were detached by incubating with 0.25% trypsin (FUJIFILM Wako Pure Chemical Corporation) for 5-10 min at 37°C and reseeded. Genomic DNA (gDNA) was isolated using ISOGENOME reagent according to the manufacturer’s instructions and stored at 4°C until use; A260/280 ratios were approximately 1.8-2.0.

Caco-2 cells were selected as a well-established human intestinal epithelial model that can be maintained under standardized culture conditions [[14], [15], [16],^17^]. Because the objective of this study was to validate the Relay-MSRE-qPCR workflow rather than to model a disease phenotype, the present validation was restricted to Caco-2 gDNA; applicability to other biological sources requires independent verification, as discussed under Limitations.

### Buffer carry-over calculation

To ensure compatibility with downstream qPCR, the expected contribution of digestion buffers to the qPCR mixture after the 5 × staged-dilution relay was calculated as follows: qPCR final = L × 0.10 + M × 0.02 + Basal × 0.004. The resulting carry-over concentrations are summarized in Table 1.

**Table 1:** Carry-over of enzyme buffer and storage components into qPCR.

**Component**	**Final concentration in qPCR**	**Units**	**Contributors**
Tris-HCl	1.256	mM	L × 0.10; M × 0.02; Basal × 0.004; enzyme-storage trace components
MgCl₂	1.212	mM	L × 0.10; M × 0.02; Basal × 0.004; enzyme-storage trace components
NaCl	1.400	mM	M × 0.02; Basal × 0.004 (L contains 0 mM NaCl); enzyme-storage trace components
DTT	0.1216	mM	L × 0.10; M × 0.02 (Basal contains 0 mM DTT); enzyme-storage trace components
KCl	0.287	mM	Enzyme-storage trace components; scaled 0.10 / 0.02 / 0.004
EDTA	0.000162	mM	Enzyme-storage trace components; scaled 0.10 / 0.02 / 0.004
BSA	0.000614	mg/mL	Vendor buffers and enzyme storage; scaled 0.10 / 0.02 / 0.004
Triton X-100	0.000090	% (v/v)	Enzyme-storage (primarily Steps 2 and 3); scaled 0.10 / 0.02 / 0.004
Glycerol	≤0.62	% (v/v)	Per-step ≤5% (v/v); qPCR final = 0.10·g_L + 0.02·g_M + 0.004·g_Basal

Note: Values reflect the 5 × staged-dilution relay. The qPCR final corresponds to 0.10 × Step-3 “Calculated” (Table 4), yielding effective contributors of L × 0.10, M × 0.02, and Basal × 0.004. Here, M denotes Takara Universal Buffer M used in Step 2. The NIPPON GENE M buffer and Takara Universal Buffer H were used only in the single-enzyme buffer-comparison controls for EcoRII and VpaK11BI (AvaII), respectively, and were not part of the staged relay (Takara Basal → Takara M → Takara L).

Abbreviations: BSA, bovine serum albumin; DTT, dithiothreitol; EDTA, ethylenediaminetetraacetic acid; MgCl₂, magnesium chloride; qPCR, quantitative polymerase chain reaction.

### Assay design and primer selection

Primer design was performed using an in-house Python/Primer3 pipeline. Genomic loci were scanned in 100-bp windows (GC ≥ 50%; observed/expected CpG ratio ≥ 0.60). Candidate amplicons (130-200 bp) were required to contain at least one relevant MSRE recognition site. Primers were positioned to avoid overlap with restriction sites and amplicon termini. Three sequence-defined validation amplicons were used to benchmark the staged digestion and qPCR performance (Table 2). The complete sequence and the exact position of their cognate MSRE recognition sites are provided in Supplementary Table S1, enabling independent reconstruction of the validation assays. Common design constraints included an amplicon length of 130-200 bp; primer melting temperature (Tm) ≈ 60°C with ΔTm ≤ 1°C; GC content of 40-60%; and avoidance of long homopolymers and stable secondary structures. When a restriction site within an amplicon was unmethylated, digestion reduced amplifiable template and resulted in a measurable ΔCt change.

**Table 2:** Motif-targeted primer set aliases and sequences.

**Alias**	**Sequence (5′→3′)**	**Tm (°C)**	**Amplicon size (bp)**	**Motifs in amplicon**
Amplicon 1 F	GTGAGTGTGAGCCCAGACAA	59.89	197	EcoRII; HpaII; HhaI
Amplicon 1 R	TACGGAAAGCTGAGGTGCTG	60.04		
Amplicon 2 F	TGTGCGAGAGACACAGTGAG	59.69	133	VpaK11BI; HpaII; HhaI
Amplicon 2 R	GGCCTTGTATTCCGTGAGCT	60.11		
Amplicon 3 F	ACAAGGGCTTGAGTGGATGG	59.96	181	Eco52I; EcoRII
Amplicon 3 R	TCACACTGTGTCTCTCGCAC	59.97		

Notes: (i) “Motifs in amplicon” denotes the restriction-enzyme recognition sites present within each PCR product. (ii) Primer-placement rules ensure that cutting within the amplicon, when unmethylated, yields a measurable ΔCt increase, while off-target (“star”) activity is minimized by maintaining each digestion near its intended buffer composition and by limiting cumulative carry-over through the 5 × staged-dilution relay.

Abbreviations: bp, base pairs; F, forward primer; R, reverse primer; Tm, melting temperature; ΔCt, delta cycle threshold.

### Restriction enzyme digestion

Two digestion modes were used: (i) a sequential Relay-MSRE-qPCR multi-enzyme protocol and (ii) single-enzyme comparator and buffer-comparison conditions. For the relay workflow, Step 1 was initiated with 50-200 ng gDNA in a 20.0 μL reaction. Enzyme amounts were 1 U Eco52I in Step 1; 5 U each of HhaI, EcoRII, and VpaK11BI (AvaII) in Step 2; and 1 U HpaII in Step 3 (Table 3). Reactions were incubated at 37°C for 120 min, followed by heat-inactivation at 80°C for 20 min. After each inactivation, 4.0 μL of the reaction was transferred to a new tube and combined with 2.0 μL of the next 10 × buffer, the enzyme(s) specified in Table 3, and nuclease-free water to a final volume of 20.0 μL. Per-step glycerol was maintained at ≤5% (v/v) (Table 4). Controls included undigested input and no-enzyme controls.

Single-enzyme comparators and buffer-comparison controls were performed in parallel. The comparators were run under the corresponding buffer conditions used in the buffer-comparison experiments (20.0 μL total; 37°C for 120 min; 80°C for 20 min) using the same DNA input range, followed directly by qPCR. Takara Universal Buffer M was selected as the common Step-2 buffer for HhaI, EcoRII, and VpaK11BI (AvaII). Its suitability was evaluated experimentally by comparing the NIPPON GENE M buffer with Takara Universal Buffer M for EcoRII, and Takara Universal Buffers H and M for VpaK11BI (AvaII) (Fig. 1). Table 3 sets out the CpG versus non-CpG recognition logic, including the CpG overlap within each motif and the blocked 5-mC position(s). HpaII, HhaI, and Eco52I provide CpG-dependent readouts, whereas EcoRII and VpaK11BI serve as auxiliary sentinels for non-CpG contexts and for stress-testing buffer rebuilds across distinct optimal conditions (Table 3).

**Fig. 1:**
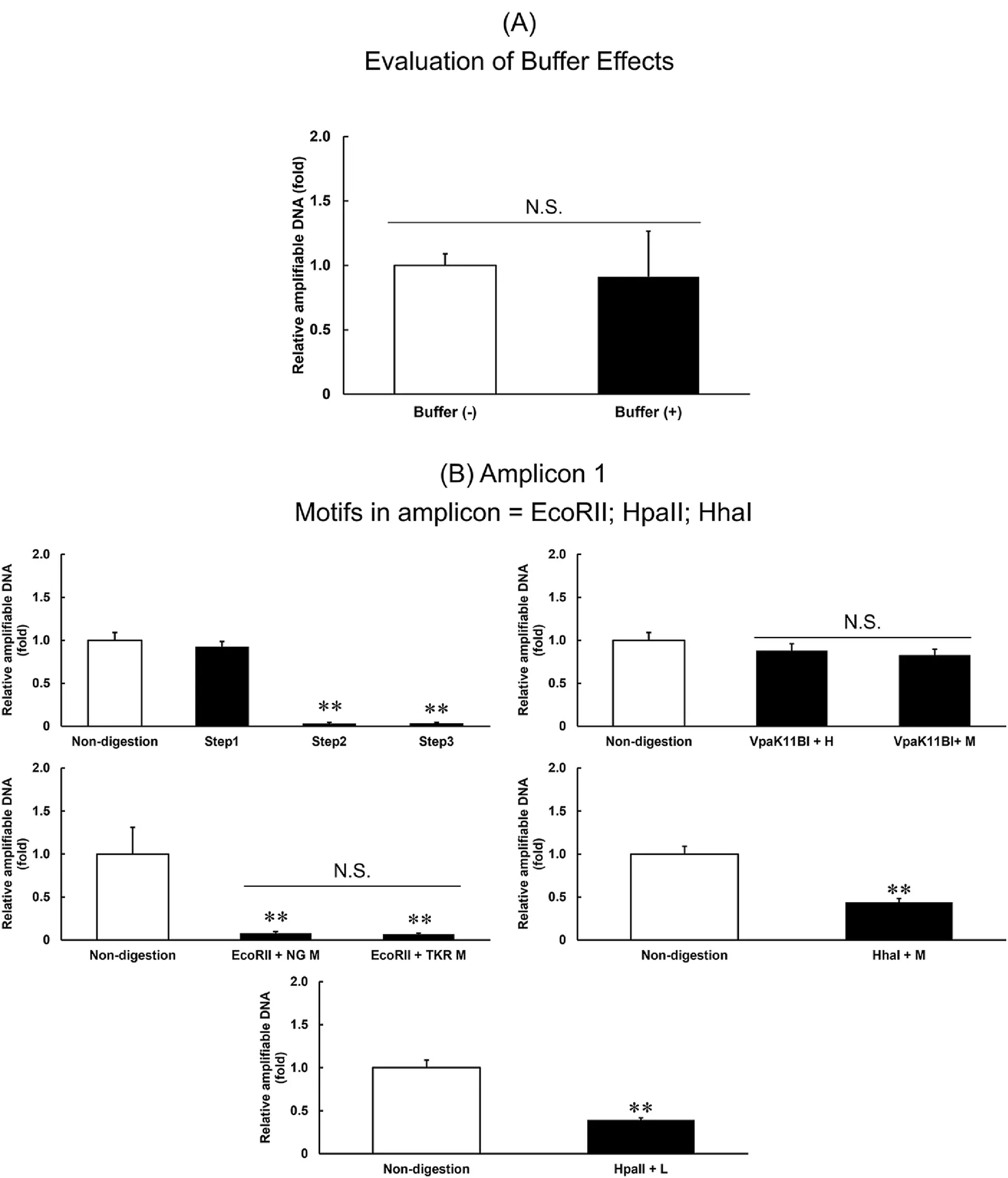

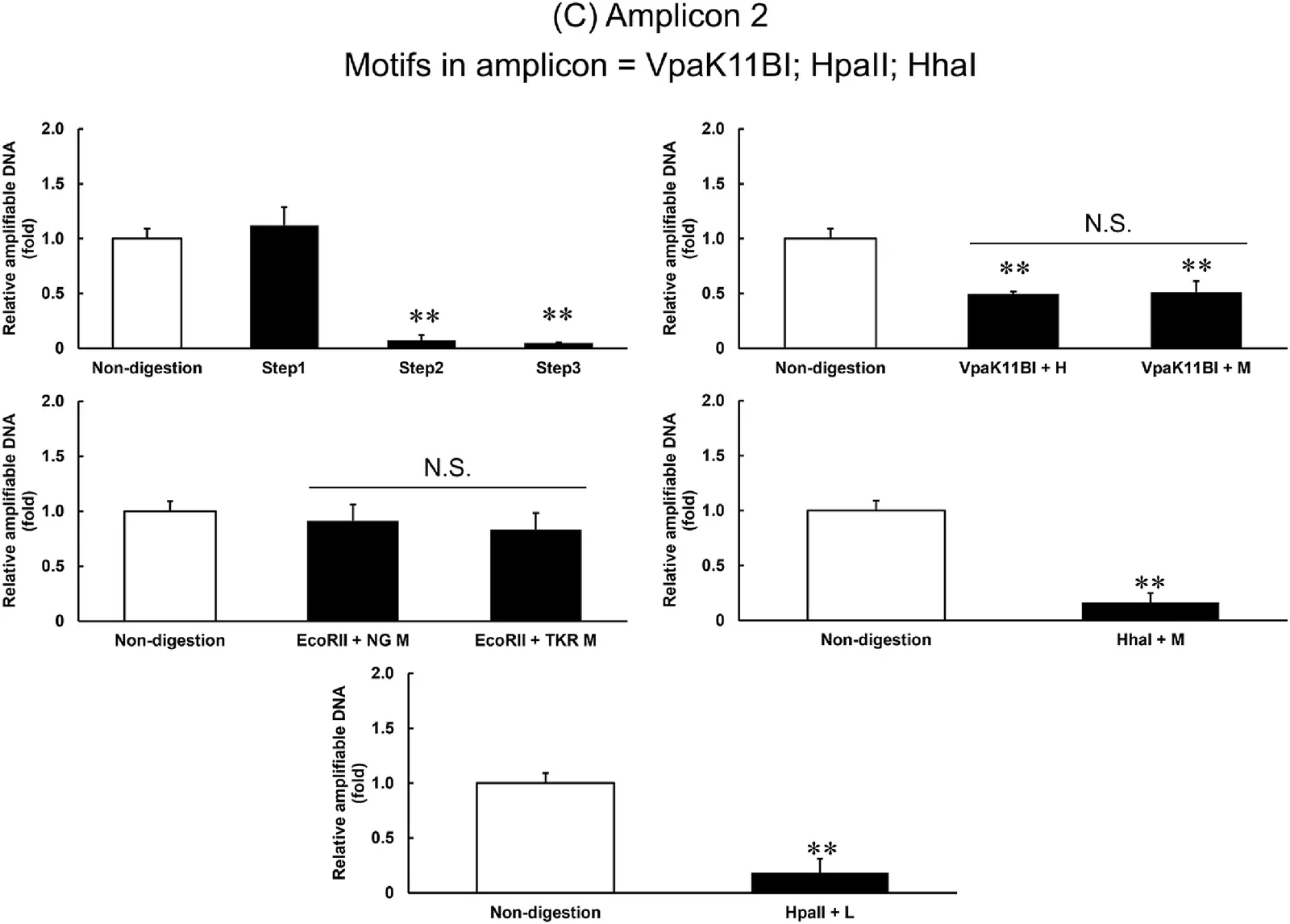

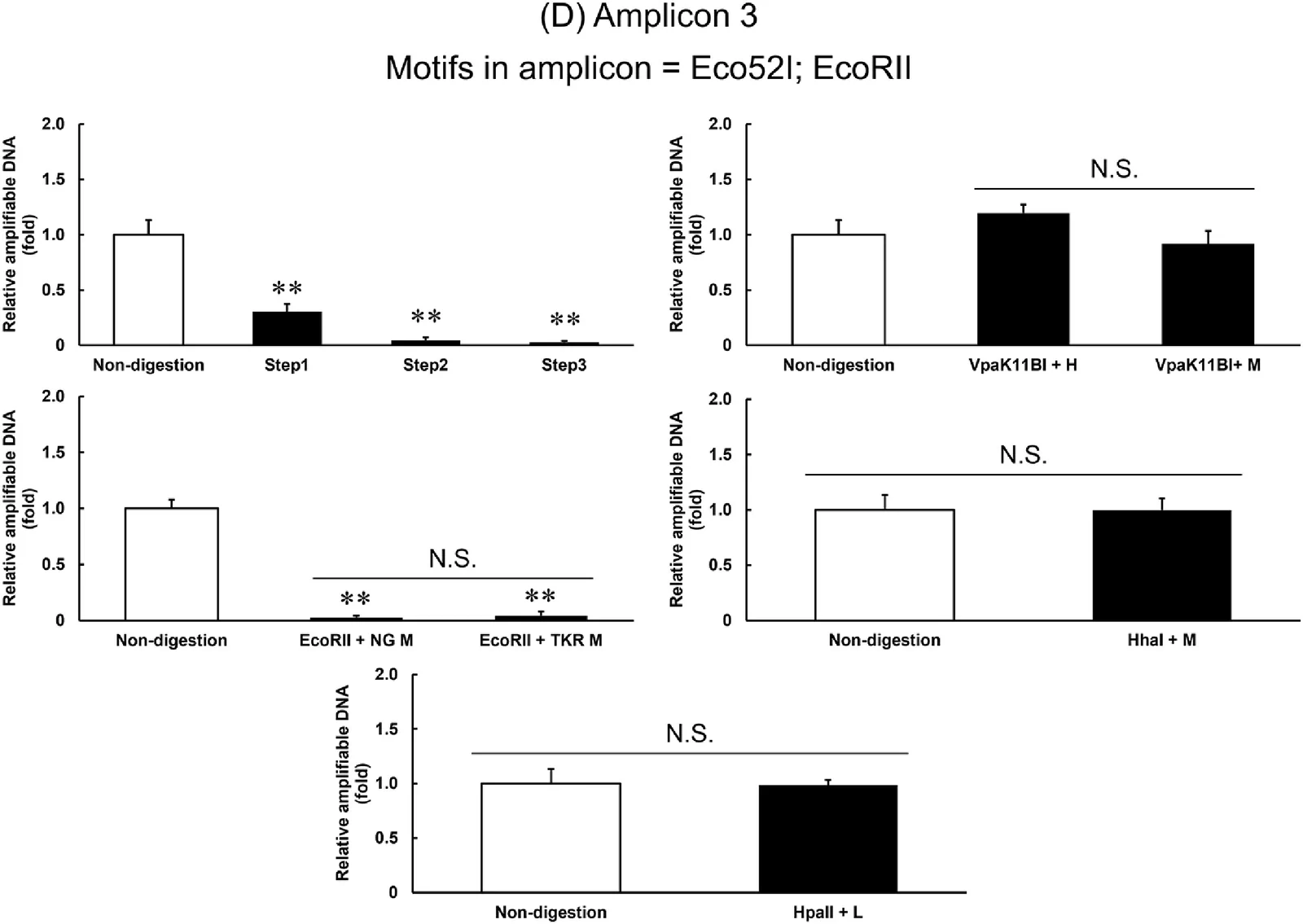
qPCR validation under relay and comparator conditions. (A) Buffer-only controls (no enzyme). Ct values were converted to copy numbers using a standard curve and expressed as relative amplifiable DNA (fold), with Buffer (-) used as a reference. Buffer (+) did not differ significantly from Buffer (-). (B-D) Amplicons 1-3. Relative amplifiable DNA under the relay sequence (Steps 1-3) was compared with that obtained under the corresponding single-enzyme comparator conditions. Digestion at cognate restriction sites reduced the amount of amplifiable DNA, whereas non-cognate enzyme conditions showed no significant reduction. Buffer-comparison controls are shown for EcoRII (NIPPON GENE M versus Takara Universal Buffer M) and VpaK11BI (AvaII) (Takara Universal Buffer H versus M), with no significant differences observed between the respective buffer conditions. Data are mean ± SD (n = 3 independent digestion workflows). Two-group comparisons used Student’s *t*-test; ≥3 groups used one-way ANOVA followed by Tukey’s multiple-comparison test. Significance is indicated relative to the corresponding control (Panel A, Buffer [-]; Panels B-D, Non-digestion): *p < 0.05, **p < 0.01; N.S., not significant.

Abbreviations: Ct, cycle threshold; ANOVA, analysis of variance; SD, standard deviation; N.S., not significant; NG M, NIPPON GENE M buffer; H, Takara Universal Buffer H; M and TKR M, Takara Universal Buffer M.

To directly evaluate the cleavage of unmethylated substrates, PCR-generated Amplicons 1-3 were used as defined unmethylated controls. Amplicon 1 was digested separately with EcoRII, HhaI, or HpaII; Amplicon 2 with VpaK11BI (AvaII); and Amplicon 3 with Eco52I (XmaIII). Matched no-enzyme reactions were processed in parallel. Digestions were performed under the corresponding buffer conditions used in this study and were followed by qPCR with the same primer pairs. The remaining amplifiable DNA was expressed relative to the matched non-digestion control, which was normalized to 1.0 (Supplementary Fig. S1).

Planned relay order (buffers rebuilt to 1 × at each stage):

Step 1: Eco52I in Basal → heat inactivate → ≥5 × staged-dilution → rebuild Takara M buffer
Step 2: HhaI + EcoRII + VpaK11BI (AvaII) in Takara M → heat inactivate → ≥5 × staged-dilution → rebuild L buffer
Step 3 (final): HpaII in L → heat inactivate → qPCR

**Table 3:** Methylation-sensitive restriction enzyme (MSRE) panel used in this study.

**Enzyme**	**Recognition sequence**	**Methylation sensitivity (summary)**	**Working conditions (per 20 μL)**	**Heat inactivation**
Eco52I (XmaIII)	CGGCCG	Blocked by CpG methylation within the motif	Basal 1 ×; 1 U; 0.01% BSA; 37°C 120 min	80°C, 20 min
HhaI	GCGC	Blocked by CpG methylation (GmCGC)	M 1 ×; 5 U; 37°C 120 min	
EcoRII	CCWGG (W = A/T)	Blocked by Dcm-type 5-mC at CCWGG	Takara M 1 × (relay); NIPPON GENE M 1 × (comparator); 5 U; 37°C 120 min	
VpaK11BI (AvaII)	GGWCC (W = A/T)	Methylation effects depend on cytosine context	M 1 ×; 5 U; 37°C 120 min	
HpaII	CCGG	Blocked by 5-mC at the inner C (CmCGG); sensitive to CpG methylation	L 1 ×; 1 U; 37°C 120 min	

Notes: The 10 × buffers used in the relay were obtained from Takara Bio Inc. and rebuilt to 1 × for each step: Eco52I Basal buffer for Eco52I; Universal Buffer M for HhaI / EcoRII / VpaK11BI (AvaII); and Universal Buffer L for HpaII. Enzyme volumes (v_enzyme_) were adjusted such that glycerol content did not exceed 5% (v/v) per step. The NIPPON GENE M buffer supplied with EcoRII was used only for the single-enzyme buffer-comparison experiment.

Abbreviations: 5-mC, 5-methylcytosine; CpG, cytosine-phosphate-guanine dinucleotide; MSRE, methylation-sensitive restriction enzyme; v_enzyme_, enzyme volume; Basal, L, M, and H, Takara restriction-enzyme buffers.

**Table 4:** Flow of enzyme-buffer carry-over through the three steps of the relay.

**Step**	**Component**	**Target (mM)**	**Calculated (mM)**	**Δ (mM)**
**Step 1 (Basal 1 ×)**	Tris-HCl	10.00	10.05	0.05
	MgCl₂	3.00	3.00	0
	NaCl	100.00	100.00	0
	DTT	0	0.005	0.005
**Step 2 (Takara M 1 × + Step 1 / 5)**	Tris-HCl	10.00	12.76	2.76
	MgCl₂	10.00	10.60	0.60
	NaCl	50.00	70.00	20.00
	DTT	1.00	1.08	0.08
**Step 3 (L 1 × + Step 2 / 5)**	Tris-HCl	10.00	12.56	2.56
	MgCl₂	10.00	12.12	2.12
	NaCl	0	14.00	14.00
	DTT	1.00	1.22	0.22

Notes: “Calculated” values were calculated employing the formula: 4.0 μL carry-over + (14.0 − v_enzyme_) μL water + 2.0 μL next 10 × buffer + v_enzyme_ μL enzyme, with the Takara 1 × buffer formulations used in the relay (Basal, M, and L) and the digestion order above. Minor contributions from enzyme-storage solutions (e.g., NaCl, MgCl₂, Triton X-100, DTT, EDTA, BSA) are included in the totals. Per-step glycerol was maintained at ≤5% (v/v). In the L buffer, NaCl target = 0 mM; thus, percent deviation is undefined, and absolute carry-over (14.00 mM) is reported. For correspondence with Table 1, qPCR final = 0.10 × Step-3 “Calculated” values (e.g., Tris-HCl 12.56 → 1.26 mM; MgCl₂ 12.12 → 1.21 mM; NaCl 14.00 → 1.40 mM; DTT 1.22 → 0.12 mM). Despite these deviations, qPCR performance criteria—single-peak melt curves, efficiency 90-110%, and ΔCt behavior—were maintained.

Abbreviations: BSA, bovine serum albumin; DTT, dithiothreitol; EDTA, ethylenediaminetetraacetic acid; MgCl₂, magnesium chloride; qPCR, quantitative polymerase chain reaction; ΔCt, delta cycle threshold; v_enzyme_, enzyme volume.

### Quantitative polymerase chain reaction (qPCR)

qPCR was performed as previously reported [[14], [15], [16], [17], [18], [19]], with minor standardizations, on an Applied Biosystems 7500 Fast instrument using KAPA SYBR® FAST Master Mix. Unless otherwise stated, reaction volumes were 10.0 μL and contained 1.0 μL of a Step-3 digest (carry-over coefficient f = 0.10). A typical reaction comprised 5.0 μL of 2 × master mix (1 × final), 0.20 μM of each primer, 1.0 μL of the Step-3 digest, and nuclease-free water to 10.0 μL. Cycling conditions were as follows: 95°C for 5 min; 40 cycles of 95°C for 5 s, 60°C for 34 s, and 72°C for 1 s; followed by melt-curve analysis (60°C for 20 s, ramping to 95°C at 0.1°C s⁻¹). Assay design and validation conformed to the MIQE guidelines [3]. No-template and undigested controls were included in each run. Relative methylation-sensitive readouts were expressed as ΔCt values between digested and undigested aliquots.

### Agarose gel electrophoresis

gDNA digests were analyzed on 1.2% (w/v) agarose gels prepared in 1 × Tris-borate-EDTA (TBE) buffer, using SAFELOOK™ Pre-Red pre-loading stain at a final concentration of 1 × . Typical DNA loads per lane were 500 ng (Step 1), 100 ng (Step 2), and 20 ng (Step 3). Electrophoresis was performed at 5-7 V cm⁻¹ for 40-60 min, and gels were imaged under blue-light illumination using a ChemiDoc™ imaging system (Bio-Rad Laboratories, Inc., CA, USA). A KAPA Express DNA Ladder (KAPA Biosystems; 100, 200, 400, 800, 1600, 4000, and 8000 bp) was used for fragment-size estimation. Under the 5 × relay workflow, equal-volume loading produces a nominal mass ratio of Step 1:Step 2:Step 3 ≈ 1:0.2:0.04. Consequently, later-step lanes appear fainter unless mass-normalized. For qualitative visualization of progressive fragmentation, equal-volume loading was used by default; when indicated, proportional mass-normalization was applied to later steps. Within each gel, exposure and acquisition settings were kept constant across all lanes.

### Statistical analyses

All statistical analyses were performed using BellCurve for Excel, version 4.10 (SSRI Co., Ltd., Tokyo, Japan). Differences between two groups were evaluated using Student’s *t*-test, and differences among more than two groups were evaluated using one-way analysis of variance (ANOVA) followed by Tukey’s multiple-comparison test. Statistical significance was set at *p* < 0.05 or *p* < 0.01. For the defined unmethylated-control experiments, the results of which are presented in Supplementary Fig. S1, each enzyme-treated condition was compared separately with the corresponding non-digestion control using Student’s *t*-test.

## Method validation

### Quantification and fold-change framework

Ct values were converted to copy numbers using standard curves, and group differences were evaluated as fold-change (mean ± SD, n = 3). The analytical framework followed previously described equivalence and buffer-compatibility approaches [20,21], and all reporting elements adhered to the MIQE 2009 and MIQE 2.0 guidelines [3,11]. In buffer-only controls (without restriction enzymes), the Buffer (+) condition was indistinguishable from Buffer (−), indicating that the buffer conditions used in the relay did not measurably inhibit amplification (Fig. 1A). This observation is consistent with the carry-over accounting summarized in Table 1 and the Step-3 operational mixture described in Table 4, confirming that salts, Mg²⁺, glycerol, bovine serum albumin (BSA), and any nonionic surfactants remained within SYBR qPCR-tolerable limits [1,3,10]. Standard curves (≥5 points; 10-fold serial dilutions) yielded efficiencies of 90-110% and *R*² ≥ 0.99. Melt-curve profiles were single-peaked with no primer-dimer formation in no-template controls (NTCs; data not shown). Unless otherwise specified, results represent three biological replicates (n = 3), each comprising one independent restriction-digestion workflow followed by a single qPCR measurement.

### Equivalence to single-enzyme digestion and buffer comparison

For each amplicon, fold-change values obtained from the staged-dilution sequence (Steps 1-3) closely matched those obtained under the corresponding single-enzyme comparator conditions. Buffer-comparison controls showed no significant difference between the NIPPON GENE M and Takara Universal Buffer M conditions for EcoRII in Amplicon 1 (*p* = 0.539) or Amplicon 3 (*p* = 0.497). VpaK11BI (AvaII) also showed no significant difference between Takara Universal Buffers H and M (p = 0.789), whereas HhaI retained the expected site-specific digestion response in Takara Universal Buffer M (Fig. 1B-D). These results support Takara Universal Buffer M as the common Step-2 reaction environment for HhaI, EcoRII, and VpaK11BI under the tested Relay-MSRE-qPCR conditions. They also show that staged-dilution buffer rebuilding preserves quantitative behavior equivalent to that in single-step digestion while tolerating the tested buffer differences [20,21].

### Per-site responsiveness within products

Within each amplicon, only digestion conditions corresponding to cognate recognition sites produced significant ΔCt changes relative to the undigested or buffer-only baseline (Fig. 1B-D). For Amplicon 1, *p*-values were 0.00680 and 0.00652 for EcoRII in NIPPON GENE M and Takara Universal Buffer M, respectively; 5.87 × 10⁻⁴ for HhaI; and 3.32 × 10⁻⁴ for HpaII. For Amplicon 2, *p*-values were 7.47 × 10⁻⁴ and 0.00345 for VpaK11BI in H and M buffers, respectively; 3.12 × 10⁻⁴ for HhaI; and 8.57 × 10⁻⁴ for HpaII. For Amplicon 3, *p* = 0.00131 for the Eco52I-containing Step-1 condition and *p* = 3.12 × 10⁻⁵ and 4.48 × 10⁻⁵ for EcoRII in NIPPON GENE M and Takara Universal Buffer M, respectively. Consistent with these native-DNA responses, digestion of defined unmethylated PCR amplicons markedly reduced the remaining amplifiable template for all five enzymes (Supplementary Fig. S1). Relative amplifiable DNA after digestion was 0.035-fold for EcoRII, 0.247-fold for HhaI, and 0.296-fold for HpaII in Amplicon 1; 0.080-fold for VpaK11BI in Amplicon 2; and 0.136-fold for Eco52I in Amplicon 3, in each case relative to the matched non-digestion control normalized to 1.0. These results directly demonstrate substantial cleavage of unmethylated substrates containing the recognition sites under the tested conditions.

### Gel corroboration and loading controls

Agarose gel electrophoresis independently corroborated staged digestion under fixed exposure conditions. With equal-volume loading, lane intensities decreased from Step 1 to Step 3, consistent with the planned 5 × staged-dilution series, and the staged-digestion lanes showed progressive fragmentation from gDNA through Steps 1-3 (Fig. 2). An undigested gDNA lane served as the high-molecular-weight reference. Buffer-comparison controls—EcoRII in NIPPON GENE M versus Takara Universal Buffer M, and VpaK11BI (AvaII) in Takara Universal Buffer M versus H—produced indistinguishable smear profiles within normal gel-to-gel variation (Fig. 2A), consistent with the qPCR results in Fig. 1B-D. Together with buffer-only qPCR controls (Fig. 1A), these data demonstrate that the observed ΔCt changes arise from true stepwise digestion rather than artifacts of loading, imaging, or exposure.

**Fig. 2:**
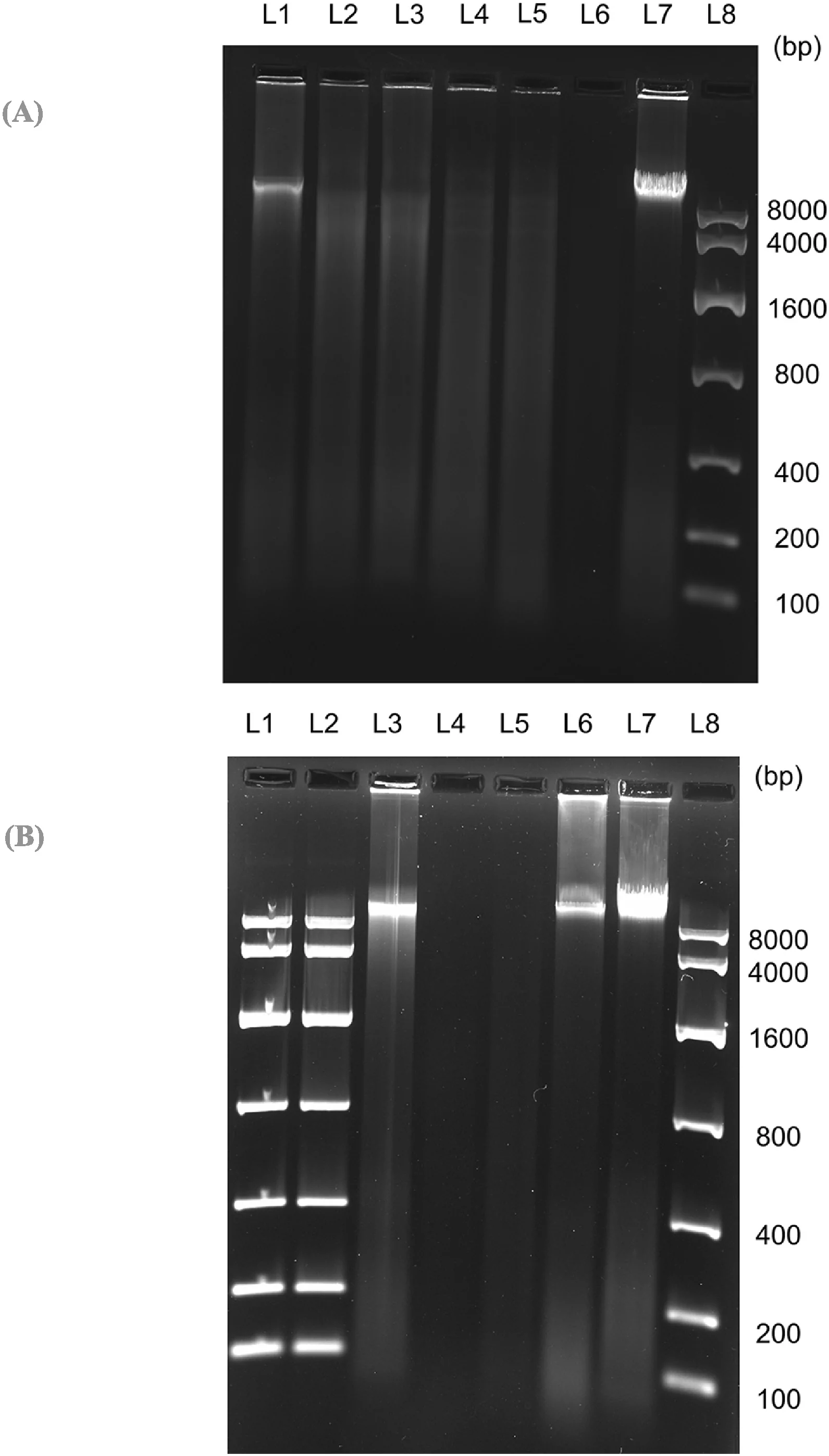
Agarose gel verification of staged restriction digestion and buffer effects. Gels were run on 1.2% (w/v) agarose in 1 × TBE buffer using SAFELOOK™ Pre-Red as a pre-loading stain (1 × final concentration) and imaged on a ChemiDoc™ system (Bio-Rad) under blue-light illumination. The KAPA Express DNA Ladder (100, 200, 400, 800, 1600, 4000, and 8000 bp) was used; molecular sizes corresponding to the ladder are indicated adjacent to the marker lanes. **(A) Single-enzyme digests in different buffers and Step-2 product.** Lanes: 1, HhaI + M; 2, VpaK11BI (AvaII) + M; 3, VpaK11BI + H; 4, EcoRII + TKR M; 5, EcoRII + NG M; 6, Step-2 product (M); 7, genomic DNA (undigested); 8, DNA marker. EcoRII showed comparable smear profiles in TKR M and NG M, while VpaK11BI showed comparable profiles in M and H; HhaI also showed strong activity in M. Lanes 1-7 each contained 100 ng DNA. **(B) Staged-digestion products across steps and HpaII control.** Lanes: 1, DNA marker; 2, DNA marker; 3, HpaII + L (single-enzyme control matching Step-3 conditions); 4, Step-3 product; 5, Step-2 product; 6, Step-1 product; 7, genomic DNA (undigested); 8, DNA marker. The staged-digestion lanes show progressive fragmentation from gDNA through Steps 1-3. Equal-volume loading under the 5 × relay yields a nominal mass ratio of Step-1:Step-2:Step-3 ≈ 1:0.2:0.04 (e.g., 500, 100, and 20 ng per lane, respectively). Exposure and acquisition settings were held constant within each gel. See also Fig. 1 for the qPCR validation of site-specific digestion under the same relay and buffer-comparison conditions.

Abbreviations: TBE, Tris-borate-EDTA; gDNA, genomic DNA; BSA, bovine serum albumin; nt, nucleotide; kb, kilobase; ng, nanogram.

### Carry-over management and buffer compositions

Relay restriction reactions used Takara Bio buffers (Basal, M, and L); the carry-over into the final qPCR was controlled by rebuilding each buffer to 1 × and applying a ≥5 × staged dilution between digestion steps. The calculated component concentrations are summarized in Table 1, Table 4. The following acceptance ranges were used to assess qPCR compatibility [12,13]:

• Buffering base (Tris-HCl, pH 7.4-8.0): maintained near 1 × through per-step buffer rebuilding
• Monovalent salts (Na⁺/K⁺): ≤100-150 mM total in the final qPCR input
• Mg²⁺ (as MgCl₂): 1.2-5 mM, acceptable
• Reducing agent (DTT): sub-millimolar carry-over
• Protein stabilizer (BSA): maintained below levels known to distort melt profiles [13]
• Glycerol: ≤5% (v/v) per step
• Nonionic surfactants (if present): diluted well below inhibitory concentrations

Under these conditions, buffer-only controls showed no amplification penalty in the Buffer (+) condition relative to the Buffer (−) condition (Fig. 1A). Two-fold dilution preserved ΔCt ≈ 1.00 ± 0.2, and buffer-comparison conditions for EcoRII and VpaK11BI caused no measurable change (Fig. 1B-D). These observations support the contention regarding the compatibility of the staged carry-over with downstream qPCR under the tested conditions [10,12,13].

### Star-activity sentinel behavior

Although a dedicated star-activity assay was not performed, non-cognate steps remained at baseline, the buffer-comparison controls did not show any significant difference, and two-fold template dilution preserved ΔCt ≈ 1.00 ± 0.2. These observations argue against substantial off-target cleavage under the tested conditions; however, a low-probability residual risk cannot be fully excluded [7,9].

### Interpretation and context

Overall, the validation data support Relay-MSRE-qPCR for relative methylation-sensitive analysis at predefined loci under the tested conditions. Because the formal validation was restricted to Caco-2 gDNA, application to other biological sources requires independent verification, as detailed under Limitations.

## Limitations

### Intrinsic limitations

The following constraints are inherent to enzyme-based interrogation of native DNA and define contexts in which Relay-MSRE-qPCR would be most informative and situations where sequencing-based approaches remain preferable:

1. Recognition-site dependence: Interrogation is limited to loci containing MSRE recognition motifs, restricting genome-wide generalization [[1], [2], [3]].
2. Population averaging: Readouts reflect mean methylation levels across input DNA and do not resolve allele-specific or cell-level heterogeneity.
3. No single-base resolution: Relay-MSRE-qPCR does not map methylation at the base level; therefore, the need for bisulfite- or enzymatic-conversion sequencing remains for single-cytosine profiling [8,[22], [23], [24]].
4. Validation scope: Formal method validation was performed using Caco-2 genomic DNA. As defined methylation standards spanning very low to very high fractional methylation levels were not evaluated, the absolute methylation range and cross-sample performance could not be established in the present study.

### Operational constraints and analytical scope

Relay performance depends on controlled enzyme loading, buffer composition, and carry-over. Per-step glycerol was maintained at ≤5% (v/v), and staged dilution limited the transfer of salts, Mg²⁺, DTT, BSA, and other enzyme-storage components into qPCR (Table 1, Table 4) [10,12,13]. Because MSRE readouts depend on recognition-site content and digestion efficiency, variation in enzyme or buffer lot may affect the ΔCt amplitudes. Defined unmethylated controls verified cleavage under the tested conditions, but fully methylated standards and defined methylated/unmethylated mixtures were not evaluated; therefore, ΔCt values cannot be directly interpreted as absolute methylation percentages [1,2]. Although the validation data did not show any evidence of substantial nonspecific cleavage under the tested conditions, rare star activity cannot be completely excluded [7,9].

### Positioning versus bisulfite and enzymatic alternatives

Compared with bisulfite- and enzymatic-conversion approaches, Relay-MSRE-qPCR offers rapid targeted analysis of DNA methylation without DNA conversion but lacks motif-independent and single-base resolution. Sequencing-based methods, therefore, remain preferable for genome-wide, allele-resolved, or single-cytosine analyses [[4], [5], [6],[22], [23], [24]]. MSRE-ddPCR provides an alternative highly sensitive digital readout for targeted methylation analysis but requires dedicated instrumentation [25].

### Reproducibility and transferability

Complete sequences of the three validation amplicons and the exact positions of their cognate MSRE recognition sites are provided in Supplementary Table S1, together with the enzyme amounts, reaction conditions, and carry-over calculations reported in Tables 1, 3, and 4. These data support independent reconstruction of the validated workflow. Further validation may address low-input performance, inter-operator reproducibility, and broader cross-vendor buffer testing, whereas transfer to other sample types or loci would require independent verification.

## Abbreviations

%CV - Percent coefficient of variation

5-mC - 5-Methylcytosine

ANOVA - Analysis of variance

BSA - Bovine serum albumin

CpG - Cytosine-phosphate-guanine dinucleotide

Ct - Cycle threshold

ddPCR - Droplet digital polymerase chain reaction

DTT - Dithiothreitol

EDTA - Ethylenediaminetetraacetic acid

gBlock - Synthetic double-stranded DNA fragment (Integrated DNA Technologies)

gDNA - Genomic DNA

H - Takara Universal Buffer H (high-salt variant)

L - Takara Universal Buffer L

M - Takara Universal Buffer M

MIQE - Minimum Information for Publication of Quantitative Real-Time PCR Experiments

MSRE - Methylation-sensitive restriction enzyme

qPCR - Quantitative polymerase chain reaction

SD - Standard deviation

TBE - Tris-borate-EDTA

Tm - Melting temperature

ΔCt - Delta cycle threshold (difference in Ct values between digested and undigested DNA samples)

## Ethics statements

This study used a commercially available, authenticated Caco-2 human cell line obtained from the European Collection of Authenticated Cell Cultures (ECACC). No human participants or primary human biological materials were involved; therefore, ethics approval and informed consent were not applicable.

## CRediT author statement

**Ippei Uemura:** Conceptualization, Formal analysis, Investigation, Writing - original draft, Writing - review & editing, Visualization, Funding acquisition. **Takashi Satoh:** Supervision, Writing - review & editing. **Natsuko Takahashi-Suzuki:** Conceptualization, Investigation, Supervision, Writing - original draft, Writing - review & editing.

## Declaration of generative AI and AI-assisted technologies in the manuscript preparation process

During the preparation of this work, the authors used ChatGPT (OpenAI) solely for language polishing and formatting assistance (e.g., grammar, phrasing, and consistency checks). After using this tool, the authors reviewed and edited the content as needed and take full responsibility for the content of the published article. No figures, images, or artwork were created or modified using generative AI, in accordance with the *MethodsX* editorial policy.

## Declaration of competing interest

The authors declare that they have no known competing financial interests or personal relationships that could have appeared to influence the work reported in this paper.

## Data Availability

Data will be made available on request.

## Appendix Supplementary materials

**Supplementary material *and/or* additional information.** Supplementary Table S1. Complete validation amplicon sequences and MSRE recognition sites. Supplementary Fig. S1. Digestion of defined unmethylated PCR amplicons by the MSREs used in Relay-MSRE-qPCR.
