# Supplementary material for: Relay-MSRE-qPCR: A sequential multi-buffer staged-dilution workflow for methylation-sensitive restriction enzyme qPCR
Source: MethodsX. 2026 Oct 12;17:104205. doi: 10.1016/j.mex.2026.104205 (PMC13734947; doi:10.1016/j.mex.2026.104205)
Supplement: Supplementary file 1 [file mmc1.docx]

**Supplementary Table S1. Complete validation amplicon sequences and MSRE recognition sites.**

|  | **Amplicon 1** | **Amplicon 2** | **Amplicon 3** |
| --- | --- | --- | --- |
| **Complete amplicon sequences** | GTGAGTGTGAGCCCAGACAAAAACCTCCCTGCAGGGAGGCTGAGGGGGCGGTCGCAGGTGCAGCTCACGGCCACCAGGGGGCGCGCGGAGCTCACGGAATACAAGGCCGGGTCAGGAGCAGGTGCAGGGTGAGCGGGGCTTGCTCATCTTCTCAGAGATCATCATCTCCCTCCTCGCCAGCACCTCAGCTTTCCGTA | TGTGCGAGAGACACAGTGAGGGGAGGTGAGTGTGAGCCCAGACACAAACCTCCCTGCAGGGAGGCTGAGGGGACCGGCGCAGGTGCAGCTCACGGCCAGCAGGGGGCGCGCGGAGCTCACGGAATACAAGGCC | ACAAGGGCTTGAGTGGATGGGAAGGATCATCCCTATCCTTGGTATAGCAAACTACGCACAGAAGTTCCAGGGCAGAGTCACGATTACCGCGGACAAATCCACGAGCACAGCCTACATGGAGCTGAGCAGCCTGAGATCTGAGGACACGGCCGTGTATTACTGTGCGAGAGACACAGTGTGA |
| **Internal MSRE sites** | EcoRII CCAGG at 74–78;  HhaI GCGC at 81–84 and 83–86;  HpaII CCGG at 107–110. | VpaK11BI GGACC at 71–75;  HpaII CCGG at 74–77;  HhaI GCGC at 77–80, 106–109, and 108–111. | EcoRII CCAGG at 67–71;  Eco52I CGGCCG at 147–152. |

All sequences are shown 5′ to 3′. Recognition-site coordinates are 1-based positions within the complete PCR amplicon. These sequences correspond to the validation amplicons reported in Table 2 of the revised manuscript.

| **(A)** | **(B)** |
| --- | --- |
| ** | ** |
| **(C)** | **(D)** |
| ** | ** |
| **Supplementary Figure S1. Digestion of defined unmethylated PCR amplicons by the MSREs used in Relay-MSRE-qPCR.**  PCR-generated unmethylated control amplicons were digested with the corresponding restriction enzymes and quantified by qPCR using the same primer pairs. Relative amplifiable DNA was normalized to the matched non-digestion control (1.0). (A) Amplicon 1 digested with EcoRII or HhaI; (B) Amplicon 1 digested with HpaII; (C) Amplicon 2 digested with VpaK11BI (AvaII); and (D) Amplicon 3 digested with Eco52I (XmaIII). Data are presented as mean ± SD (n = 3). Statistical comparisons were performed using Student’s *t*-test. ***p* < 0.01 versus the corresponding non-digestion control. Relative amplifiable DNA after digestion was 0.035-fold for EcoRII (***p* = 7.62 × 10⁻⁵**), 0.247-fold for HhaI (***p* = 2.52 × 10⁻⁴**), and 0.296-fold for HpaII (***p* = 1.29 × 10⁻⁴**) in Amplicon 1; 0.080-fold for VpaK11BI (***p* = 2.20 × 10⁻⁴**) in Amplicon 2; and 0.136-fold for Eco52I (***p* = 1.07 × 10⁻⁴**) in Amplicon 3, in each case relative to the matched non-digestion control normalized to 1.0. | |

Amplicon 1

|  | Relative amplifiable DNA (fold) |
| --- | --- |
| Non-digestion | 1 |
| EcoRII | 0.0347389 |
| HhaI | 0.247191 |

Amplicon 1

|  | Relative amplifiable DNA (fold) |
| --- | --- |
| Non-digestion | 1 |
| HpaII | 0.296444 |

Amplicon 2

|  | Relative amplifiable DNA (fold) |
| --- | --- |
| Non-digestion | 1 |
| VpaK11BI | 0.0799651 |

Amplicon 3

|  | Relative amplifiable DNA (fold) |
| --- | --- |
| Non-digestion | 1 |
| Eco52I | 0.136156 |
